# Effectiveness of an Internet-Based Machine-Guided Stress Management Program Based on Cognitive Behavioral Therapy for Improving Depression Among Workers: Protocol for a Randomized Controlled Trial

**DOI:** 10.2196/30305

**Published:** 2021-09-29

**Authors:** Norito Kawakami, Kotaro Imamura, Kazuhiro Watanabe, Yuki Sekiya, Natsu Sasaki, Nana Sato, SMART-CBT Project Team

**Affiliations:** 1 Department of Mental Health Graduate School of Medicine The University of Tokyo Tokyo Japan; 2 Department of Public Health Kitasato University School of Medicine Sagamihara Japan

**Keywords:** deep learning, unguided intervention, universal prevention, workplace, depression, machine learning

## Abstract

**Background:**

The effect of an unguided internet-based cognitive behavioral therapy (iCBT) stress management program on depression may be enhanced by applying artificial intelligence (AI) technologies to guide participants adopting the program.

**Objective:**

The aim of this study is to describe a research protocol to investigate the effect of a newly developed iCBT stress management program adopting AI technologies on improving depression among healthy workers during the COVID-19 pandemic.

**Methods:**

This study is a two-arm, parallel, randomized controlled trial. Participants (N=1400) will be recruited, and those who meet the inclusion criteria will be randomly allocated to the intervention or control (treatment as usual) group. A 6-week, six-module, internet-based stress management program, SMART-CBT, has been developed that includes machine-guided exercises to help participants acquire CBT skills, and it applies machine learning and deep learning technologies. The intervention group will participate in the program for 10 weeks. The primary outcome, depression, will be measured using the Beck Depression Inventory II at baseline and 3- and 6-month follow-ups. A mixed model repeated measures analysis will be used to test the intervention effect (group × time interactions) in the total sample (universal prevention) on an intention-to-treat basis.

**Results:**

The study was at the stage of recruitment of participants at the time of submission. The data analysis related to the primary outcome will start in January 2022, and the results might be published in 2022 or 2023.

**Conclusions:**

This is the first study to investigate the effectiveness of a fully automated machine-guided iCBT program for improving subthreshold depression among workers using a randomized controlled trial design. The study will explore the potential of a machine-guided stress management program that can be disseminated online to a large number of workers with minimal cost in the post–COVID-19 era.

**Trial Registration:**

UMIN Clinical Trials Registry（UMIN-CTR) UMIN000043897; https://upload.umin.ac.jp/cgi-open-bin/ctr_e/ctr_view.cgi?recptno=R000050125

**International Registered Report Identifier (IRRID):**

PRR1-10.2196/30305

## Introduction

### Background

Both depressive disorders and subthreshold symptoms of depression are major public health problems because of the high prevalence of depression and its substantial impacts in terms of distress, disability, and impaired quality of life [1,2]. Depression is also a leading cause of productivity loss, such as impaired work performance and missed days at work, in the workplace [3]. The primary prevention of depression is important to improve quality of life and the human capital of companies or organizations and the whole society [4].

Internet-based (or web-based) online stress management programs that incorporate cognitive behavioral therapy (CBT) have been shown to be effective in reducing symptoms of depression and the risk of major depressive disorder among symptomatic groups [5,6], and in the general [7] and working population [8]. Such internet-based CBT (iCBT) programs are easy for people to access and can be delivered to a large number of people at a relatively low cost compared to face-to-face or group-based CBT programs. In particular, during a pandemic, such as COVID-19, a stress management program, which does not require face-to-face contact, is highly desirable.

However, previous reviews reported a clear difference in the effects of iCBT programs with and without therapist support [9,10]. The effect sizes for improving stress, depression, and anxiety of adults were moderate (Cohen *d*=0.61-0.64) for iCBT programs guided by therapists, in which mental health specialists, such as psychologists, help participants to learn CBT skills, for instance, by responding to their questions or providing feedback on their homework in the program. On the other hand, the effect sizes were smaller (Cohen *d*=0.25-0.33) for fully automated self-guided iCBT programs without therapist support [9,10]. Moreover, in the workplace, guided interventions showed better intervention effects on the symptoms of stress and depression than unguided interventions (Hedges *g*=0.39 and 0.34, respectively) [8]. In addition, unguided iCBT interventions may be effective in reducing depression in the short term (eg, 3 months), but they sometimes fail to show intermediate-term (eg, 6 months or longer) effects [11,12]. However, a guided iCBT program requires trained therapists and thus needs more financial and human resources than an unguided program. This might limit the dissemination and implementation of CBT programs in a low-resource setting, such as small enterprises and middle- and low-income countries [13].

Concerning the target population of interventions, interventions that focus on high-risk or symptomatic populations (selective and indicated interventions) often yielded greater effects than a universal intervention [14]. For instance, among web-based psychological interventions in the workplace, the effect sizes of web-based psychological interventions were larger for indicated interventions than for universal prevention interventions (Hedges *g*=0.52 and 0.25, respectively) [8]. However, the reduction in symptoms may last for a shorter duration for selective and indicated interventions compared to universal interventions [15]. Universal interventions also have an advantage in possessing a less stigmatizing nature than other interventions in the field of mental health [16]. Thus, universal preventive interventions are recognized as a potentially desirable approach [17]. If we successfully enhance the effectiveness of this approach, a universal prevention intervention would be a more promising approach for the prevention of depression in the community or workplace.

A possible strategy to improve the effect of a fully automated unguided iCBT program is to incorporate an automated function to support users’ learning in an unguided program that is equivalent to therapist-guided ones by applying programmed interactions between a user and the system, for instance, individualized feedback regarding the assessment of stress and mental health status to the user, a step-by-step guide to develop the CBT skills of the user, suggestions regarding options available to the user, and advice on homework done by the user to improve his/her skills. Automated machine algorithms for these functions could be developed applying a machine-learning prediction based on a large data set, a scenario-based chatbot guide, and a deep learning technology to evaluate the appropriateness of a user’s responses in his/her homework exercises. To date, no such “machine-guided” iCBT program has been tested for its effectiveness in alleviating depression among workers.

### Objectives

To address these unresolved issues, our randomized controlled trial (RCT) aims at investigating the effectiveness of a fully automated machine-guided iCBT program supported by artificial intelligence (AI) technologies, called “SMART-CBT,” for subthreshold symptoms of depression (the primary outcome) at 3- and 6-month follow-ups and for secondary outcomes (psychological distress, fear of COVID-19, and sick leave days at 3- and 6-month follow-ups, and major depressive episodes [MDEs] over a 12-month period) compared to treatment as usual (TAU). Participants will be healthy workers selected from the general working population (universal prevention). We hypothesize that this fully automated machine-guided program will improve the symptoms of depression compared with the control group, with an equal or greater effect size as in previous guided web-based psychological interventions at the 6-month follow-up (0.16-0.25) [8,18] in the total sample (universal prevention). We will also investigate the effect on depression among participants with subthreshold depression (indicated intervention).

## Methods

### Trial Design

The study will be a nonblinded, parallel, two-arm RCT with a single intervention group that will receive the SMART-CBT intervention and a control group that will receive TAU. The allocation ratio of the intervention group to the control group is 1 to 1. Participants will be randomly allocated to either the intervention group or the control group after they have completed a baseline online questionnaire. Follow-up surveys will be conducted at 3, 6, and 12 months after the baseline measurement, using online questionnaires. The study protocol has been registered at the UMIN Clinical Trials Registry (UMIN-CTR; ID UMIN000043897). This protocol manuscript has been reported according to the SPIRIT (Standard Protocol Items: Recommendations for Interventional Trials) guideline checklist [19].

### Participants

The target population of this RCT will be healthy full-time workers from the general working population, because our intention is to develop a “generic” internet-based stress management program that is effective and can be disseminated to workers across industries and occupations. Full-time employees currently working in companies/organizations in Japan will be invited to participate in the study through an internet survey company (Macromill Inc). The inclusion criteria are as follows: (1) adult (age 20-60 years), (2) full-time employee, and (3) access to the internet via a PC, smartphone, or tablet. The exclusion criteria are as follows: (1) not on long-term sick leave or maternity/childcare leave or not temporally laid off at the time of recruitment, (2) no sick leave of 15 days or more in the past 3 months, (3) not receiving treatment from mental health professionals, (4) no MDE in the past month (based on the self-report Mini-International Neuropsychiatric Interview [MINI]), and (5) not a business owner, self-employed individual, freelancer, or part-time worker.

### Procedure

Participants will be recruited from a pool of 300,000 people living in all 47 prefectures of Japan who registered with an online survey company. A clinical research coordinator (CRC) from the survey company will send an invitation to this population, with a description of the study’s aim and procedure, and obtain online informed consent to participate in the study. We will recruit 1600 participants in total, including 200 males and 200 females in each of four age groups (20-29, 30-39, 40-49, and 50-60 years old). The CRC will ask those who agree to participate in the study to complete an online questionnaire for the baseline survey. We expect that at least 1400 individuals will be eligible based on the inclusion and exclusion criteria. These participants will be randomly allocated to either the intervention group (n=700) or the control group (n=700). Participants in the intervention group will be asked to use the intervention program for 6 weeks after the baseline survey, and they will be encouraged to revisit and review the program for an additional 4 weeks. Participants in the control group will be given a chance to receive the intervention program upon completion of the study. The participants in the intervention group will be given a token equivalent to JPY 1000 (USD 9.1) if they complete all modules of the program. The participants in the control group will be given a token equivalent to JPY 100 (USD 0.91). Participants in both groups will be given a token equivalent to JPY 30 (USD 0.27) for completing each of the surveys (baseline, and 3-, 6-, and 12-month follow-up surveys).

### Intervention

The SMART-CBT program is a 6-week web-based training course to provide CBT stress management skills, accessed by using a URL through a PC or smartphone via the internet (Multimedia Appendix 1). We did not develop a special app for this program. The program is structured as six modules, with one module given per week. Each module consists of a lecture of about 10 minutes, followed by a module-specific exercise (Table 1). In addition, a seventh module (a lecture only) was developed to address coping with psychological stress due to the COVID-19 pandemic, considering that the study will be conducted while the pandemic is still ongoing. Unlike the other modules, participants may access this COVID-19–specific module any time after they start the program. Participants in the control group, as well as the intervention group, will be able to access in-house occupational health services or mental health services from an outside-company employee assistance program, through arrangements made by their companies (TAU condition).

**Table 1:** The structure and content of the machine-guided internet-based stress management program applying artificial intelligence technology based on cognitive behavioral therapy (SMART-CBT).

Module number	Module theme	Lecture (number of webpages)	Exercise (applied computational technologies)
Module 1	The stress model and its components	Concepts of stressors and stress reactions; specific examples of stressors and stress reactions (six pages)	Self-assessment of the levels of job stressors and psychological stress reactions (machine-learning predictions using linear and logistic regressions)
Module 2	The case formulation with the five-part model of CBT^a^	Five-part model of CBT; explanation of each component using a fictitious sample case (five pages)	To list components of the five-part CBT model based on own experience or a fictitious case (button-based bot and deep learning)
Module 3	Behavior activation and relaxation	Theory of behavior activation; practical tips to plan active behaviors; and breathing techniques for relaxation (five pages)	(1) To list candidate behaviors related to feeling better; (2) to select behaviors based on the effect and feasibility; and (3) to make a plan for behavior activation (button-based bot with a prefixed scenario)
Module 4	Cognitive restructuring: (1) awareness of the association between thoughts and mood	Theory of cognitive restructuring; understanding of the theory with two fictitious cases; schemas; and how to identify thoughts behind moods (five pages)	(1) To describe events, thoughts, and moods; and (2) to associate thoughts and moods (button-based bot and deep learning)
Module 5	Cognitive restructuring: (2) changing mood by balanced thinking	Finding evidence that supports the thought and that does not support the thought; creating balanced thinking; and monitoring changing mood (six pages)	(1) To describe events, thoughts, and moods; (2) to associate thoughts and moods; (3) to find evidence that supports the thought and that does not support the thought; (4) to create balanced thinking; and (5) to confirm improved mood (button-based bot and deep learning)
Module 6	Problem solving skills	Psychological theory of problem solving; problems finding and problems shaping; listing possible solutions; and review of the outcome of an implemented solution and improve the solution (eight pages)	N/A^b^
Module COVID-19 (accessible anytime)	Coping with stress due to COVID-19	Stress due to COVID-19 outbreaks; introduction of stress management techniques (behavioral activation and cognitive restructuring); keeping a healthy lifestyle; and seeking help if needed (four pages)	N/A

^a^CBT: cognitive behavioral therapy.

^b^N/A: not applicable; no exercise is available for the module.

#### Modules

Each of the six modules was developed for a specific learning goal as follows: the stress model and its components in Module 1; case formulation with the five-part model of CBT in Module 2 [20]; behavior activation in Module 3 [21,22], with a brief introduction to relaxation [23]; cognitive restructuring involving awareness of the association between thoughts and moods in Module 4; cognitive restructuring involving changing mood through balanced thinking in Module 5 [24,25]; and problem solving skills in Module 6 [26,27]. These topics are adopted from our previous iCBT stress management program [18]. A seventh module is included concerning stress and coping with stress due to COVID-19.

#### Lectures

Each lecture is text-based, consisting of six to seven pages including basic information about a topic, guided by a dialogue among a psychology counselor, a healthy employee as a client, and an AI avatar, Mr Smart, representing the system. No audio or video lecture is used. These lectures were developed based on our previous iCBT stress management program [18]. The lecture in the COVID-19 module consists of four pages providing information about stress profiles and lifestyle changes during the COVID-19 pandemic, and introduces basic skills for improving mental health and coping with stress due to the COVID-19 pandemic, including the possible usefulness of CBT-based approaches included in other modules of the program [28], a healthy lifestyle, and help seeking [29,30].

#### Exercises

A unique feature of the program is that it includes fully automated interactive exercises on the topic of each module, following the lecture, in order to facilitate participants’ motivation to study, understand, and acquire skills relevant to the topic. Each exercise starts with a brief summary of the corresponding lecture, followed by automated interactive learning sequences guided by a predetermined scenario, linear regression prediction algorithm, or machine learning algorithm set up in the system. No exercise was prepared for Module 6 or the COVID-19 module.

##### Module 1 Exercise

For Module 1, after the lecture talks about the concepts and specific examples of job stressors, stress reactions, and long-term health outcomes, participants are asked to do a self-assessment of the levels of job stressors and psychological stress reactions. An 18-item questionnaire was developed that includes six three-item scales measuring job demands, job control, supervisor support, coworker support, and positive (vigor) and negative (depression) emotions, adopting scales and items from an already established job stress questionnaire (the Brief Job Stress Questionnaire) [31]. Five of the scales are the same as the original scales; for the sixth, a depression scale was constructed by selecting three of the six original items that showed the highest factor loadings in a factor analysis of the original scale. First, criteria were created to roughly classify participants into five groups according to each scale score using the national norm data as follows: low (<5%), low-average (5%-24%), average (25%-74%), high-average (75%-95%), and high (>95%). These criteria will be used to inform a participant of his/her relative position in terms of each scale.

Job stressors and emotional reactions have been associated with poor levels of health [32] and work performance [33]. Job stressors may also have a spillover effect on family relationships [34] and a crossover effect on the well-being of a family member [35]. Informing participants of predictions regarding these possible outcomes of one’s work and life based on the self-assessment may motivate participants to engage in the program or even reduce depression [36]. Data from a large 1-year follow-up online survey (n=2800, with a response rate of 68%) of full-time employees conducted between 2018 and 2019 was used to develop a prediction model based on the above six scales at baseline for achievement at work and personal life-related outcomes. The former was measured on a presenteeism item from the WHO Health and Work Performance Questionnaire (range 0-10) [37,38], an item simply asking about any successful event in the job (yes=1 or no=0), and an item asking whether there had been any troublesome events at work (yes=1 or no=0). Personal life–related outcome measures included items regarding subjective health status (excellent/good=1 or fair/poor=0), trouble in family relationships (yes=1 or no=0), poor relationship with family (yes=1 or no=0), and any undesirable event for a family member (yes=1 or no=0). The model was developed using linear or logistic regression to predict these outcomes at follow-up on the six scale scores, adjusting for sex and age at baseline. A sum of regression coefficients for the six scales that were statistically significant was used as an index to indicate work performance or the probability of each event 1 year later and was expressed on a 10-point scale. A personalized message will be displayed on the screen with these predictions to inform the participant what could happen in his/her work and personal life in the next year, based on the current levels of job stressors and emotional reactions.

##### Module 2 Exercise

For Module 2, which talks about the CBT case formulation, two sequential exercises have been prepared to inform participants about how to list each of the components of the five-part CBT model (event, thought, mood, behavior, and physical symptoms) [20]. In the first exercise, a case of an employee experiencing a stressful event at work is introduced. A participant is asked to classify 10 predetermined words related to the condition of this person into the most appropriate group for the four remaining components. The answer will be automatically scored (0-10 points), and he/she will be presented with another word, also with the correct answers. The participant will be encouraged to repeat this exercise until he/she scores 8 points or higher. In the second exercise, participants will be asked to fill in their own words for five boxes corresponding to the five components. Participants may do this based on their past experience or based on the case presented in the previous exercise. Once a participant completes the task, his/her responses will be automatically scored by an algorithm developed in terms of the accuracy of his/her responses.

For this module, the algorithm was developed by applying the long short-term memory (LSTM) model, one of the recurrent neural network (RNN) models that has feedback connections to deal with a series of pieces of information [39], such as a sentence including a set of words, to existing data. A large anonymous sample of 23,502 words or sentences reported for the five components by users of an online depression self-care program based on CBT was used (U2Plus, Cotree Co) [40]. The five LSTM algorithms for each component of CBT (event, thought, mood, behavior, and physical symptoms) judge whether the sentences in each box were correctly filled in. For creating supervised data, a total of 16 clinical psychologists rated each word or sentence if it included the five components according to the five-part CBT model [20]. The overall interrater reliability among the 16 clinical psychologists for the initial 1200 words/sentences was 87.3% (range 83.3%-91.3% among the pairs), and kappa was 0.714 (range 0.639-0.799 among the pairs).

For the development, validation, and evaluation of the deep learning algorithms, the supervised data were randomly divided into training data (80% of the data, N=18,801) and test data (20% of the data, N=4701), using the train_test_split from Scikit-learn. Next, the words/sentences in the training data were morphologically analyzed and counted by a Python program package, Janome [41], and the top 7500 words were coded into unique numbers. Other words were treated as out of vocabulary. The coded words were embedded into the LSTM algorithms. The sentence length was set to 20, and longer sentences were cut into multiple sentences with fewer than 20 words. Embedding size, hidden layer size, batch size, and the number of an epoch of learning were set to 32, 32, 512, and 50, respectively. A total of 20% of the training data was randomly selected and used for the validation process. Adam was adopted as an optimizer of the algorithms [42]. The learning rate was set to 0.0001. To avoid overfitting, 20% of the neural network was dropped out. Early stopping was implemented if the loss value for a given learning epoch was greater than the loss values for two previous consecutive epochs. After the validation process, the classification performance of the LSTM algorithms was tested for accuracy, sensitivity, and specificity, using the test data. The performance of the five LSTM algorithms ranged from 0.794 to 0.839 for accuracy, from 0.771 to 0.822 for sensitivity, and from 0.847 to 0.902 for specificity. All of the processes were implemented by Keras version 2.2.4 and TensorFlow version 1.14.0 in Python. An input by a participant to this exercise will be evaluated by the LSTM algorithm to be scored 0% to 100% and categorized into four groups using the sample data as a norm as follows: excellent (top 25%), good (the second 25%), moderate (25% below the average), or fair (bottom 25%). A word or phrase is highlighted for attention if it is judged by the algorithm to not be in an appropriate place. In addition, tips for categorizing the four components in the appropriate group will be shown on the screen. A participant will be encouraged to redo the exercise if his/her score is not in the top 25% and strongly encouraged to do so if the score is in the bottom 25%.

##### Module 3 Exercise

In Module 3, which talks about behavior activation, there is an exercise in which a participant can practice planning behaviors that make him/her feel better, following the basic principle of the behavioral activation treatment . First, he/she is asked to type in a box on the screen behaviors that might make him/her feel better. A total of 128 popular behaviors that might make people feel better (eg, walking and listening to music) were collected from 12 clinical psychologists, with ratings of the degree of being easily executed and the possible effect on mood for each behavior. A total of 33 behaviors not recommended for behavioral activation (eg, drinking alcohol to excess, gambling, etc) were also collected. The aforementioned large online survey asked the 2800 respondents to rate whether each of the 128 behaviors would make them feel better. A total of 40 behaviors that were endorsed by 1% or more of the respondents for men or women were selected to be part of the recommended list, because of the high diversity of the responses. These behaviors were sorted by a sum of the ratings of the degree of being easily executed and the possible effect on mood by clinical psychologists.

The list is used to help a participant to list behaviors that might make him/her feel better in the first part of the exercise, taking into account the sex of the participant. Participants will be asked to select up to three behaviors that they would like to try. Each of these behaviors will be evaluated by using an algorithm to be recommended or discouraged. This algorithm compares an input word or phrase with words or phrases in the list of recommended behaviors or that of nonrecommended behaviors. If it matches, the algorithm will show a label of being “recommended” or “avoided” for that behavior [43]. In the latter case, a message will appear to suggest reconsidering engaging in that behavior. In the next step, participants will be asked to rate each behavior on the degree of ease of execution and its expected effect on their mood using a scale from 0 to 10, and then, the system will recommend the behavior with the highest sum scores the most. Based on their preferences and the recommendation from the system, participants will select one behavior to set up a behavior activation plan, such as the date and place to execute the behavior. They will be able to save the plan to the system, and come back to review it later. An additional option function will follow-up the adherence of participants to the plan. Following the date set up to carry out the plan, the system will ask participants if they have finished the plan and how their moods have been changed by it. Based on their responses, the system will provide the participants with automatically generated comments and advise them to continue the plan or change to another one.

##### Module 4 Exercise

In Module 4, which discusses cognitive restructuring (part 1) and awareness of the association between thoughts and moods, an exercise will help participants become aware of how their thoughts and moods are associated. This exercise starts with 10 questions on schemas that may underlie maladaptive ways of thinking (such as black or white thinking, overgeneralization, etc) [22]. Participants will be asked whether these schemas apply to their own thinking. The data will be stored in the system for later use. Like the second exercise of Module 2, in this exercise, participants will be asked to enter a stressful event or situation, thoughts, and moods (three of the five components of the CBT model) into corresponding boxes on the screen; they will be permitted to do this exercise either based on their own experience or by selecting one of five fictitious cases provided by the system, or using previous saved data that they created in the second exercise of Module 2. They will also be asked to rate the extent to which they feel each presented mood on a scale from 0 to 100. The responses in the three boxes will be automatically scored by an algorithm developed by the authors to assess its accuracy, with each one being scored 0% to 100%, and categorized into four quartile groups using the sample data as a norm. Participants will be encouraged to redo the input if their score is not in the top 25%. In the next step, they will be asked to select the most relevant thought and mood if there are multiple ones. In the final step, they will be asked to think about whether the selected thought and mood are associated, in other words, if the mood would change if the thought changes. If the participants confirm the association, the exercise ends. If not, the participants will be asked to go back to the selection of thoughts and moods and redo the exercise.

##### Module 5 Exercise

In Module 5, which discusses cognitive restructuring (part 2), that is, changing mood through balanced thinking, an exercise to cover all the steps of cognitive restructuring will be presented. Participants will be asked to (1) fill in events, thoughts, and moods; (2) select one thought that is most relevant to the mood; (3) review the association between the thought and mood; (4) describe evidence that supports the thought; (5) search for evidence that does not support the thought; and (6) engage in balanced thinking and monitor changes in mood. Steps 1 to 3 use the same components of the Module 4 exercise. In step 4, participants will be asked to enter reasons that they think the thought is true (ie, evidence that supports the thought), and indicate their degree of confidence for the reason. Then, they will be asked to enter evidence that does not support the thought, which is sometimes a hard step for people.

A system was developed to help participants be aware of evidence that does not support their thoughts in three ways. First, refer to a list of 12 patterns of evidence that does not support the thought, which was developed through extensive discussion by a group consisting of clinical psychologists, mental health researchers, and master’s and doctoral students specializing in mental health, to classify patterns of 289 sets of evidence that does not support the thought extracted from data already collected from the five-part CBT case formulation reported by participants in a previous iCBT program [18]. Second, considering that the use of old sayings may help in communicating therapeutic techniques with patients and bringing about cognitive reframing in CBT [44,45], refer to a selection of 50 well-known sayings rank-ordered by the frequency of endorsement for possible effects on improving mood or reducing stress calculated from data collected via an internet survey of full-time employees (N=4120), expecting that participants may find a new way of thinking beyond their schemas by studying the philosophy underlying the saying. Third, for schemes reported by participants in the Module 4 exercise, common tips to find evidence that does not support the thought are suggested by the system, which were developed through a discussion among clinical psychologists and mental health researchers. Once words or phrases of evidence that does not support the thought are entered, the participant is asked to rate the degree of confidence for each word or phrase, using a scale from 0 to 100.

The next screen shows all the information entered about the event, the thought, the list of moods, the evidence that supports the thought, and the evidence that does not support the thought. Then, the participants will be asked to create statements that reflect balanced thinking. Tips to help participants to create balanced thinking will be provided if they request them. They will also be able to ask the system to show a sample statement of balanced thinking, which will be created by automatically connecting a word/phrase of evidence that supports the thought and a word/phrase for evidence that does not support the thought, with the highest confidence rating.

In the final step of the exercise, after participants have created the statement of balanced thinking, they will be asked to rate their mood using a scale from 0 to 100. The system automatically detects changes in mood from those entered in the mood box before, and shows an “improvement” message when mood improves. Finally, participants will be asked to rate their overall mood change using a 4-point scale (a lot, to some extent, not much, and not at all). If they rate their mood change as a lot or to some extent, the exercise will end, with a suggestion to keep the statement of balanced thinking and use it in their daily life; otherwise, they will be encouraged to try again with other evidence that does not support the thought or with an alternative statement of balanced thinking.

### Intervention Group

Participants in the intervention group will be invited to access and study the SMART-CBT program for 10 weeks in total (ie, a 6-week main study period followed by a 4-week review period) after the baseline survey. Participants in the intervention study may quit participating in or continuing the intervention solely based on their decision. During the first 6 weeks, the intervention group will be notified every Monday via email that a new module is available for the study. When participants do not complete a module by that Friday, they will receive an email reminder to encourage them to complete the module. After the initial 6 weeks, those who complete all six modules (except for the COVID-19 module) will receive a weekly email to encourage them to review the modules. If participants do not complete any module (other than the COVID-19 module), they will receive an email reminder to encourage them to review the modules. Ten weeks after the baseline survey, the intervention program will be closed.

### Control Group

Participants in the control group will not receive any intervention program during the study period. Participants in both the intervention group and the control group will be able to use an internal occupational health service and/or employee assistance program service, depending on the policy of their workplace. Participants in the control group will be provided a chance to use the intervention program after the study period.

### Outcomes

All outcomes will be measured by using online questionnaires. Nonrespondents will receive a reminder email from the research center to participate in the surveys. Outcome measures other than the occurrence of an MDE will be assessed at baseline and at two follow-up time points, 3 and 6 months after the baseline survey. MDEs will be assessed at baseline, and 6- and 12-month follow-ups. Table 2 provides an overview of the outcome measures for each survey.

**Table 2:** Outcomes to be evaluate in this study: measures and timing of measurement.

Outcome		Measure	Baseline (T1)	3-month follow-up (T2)	6-month follow-up (T3)	12-month follow-up (T4)
**Primary outcome**						
	Depression	BDI-II^a^	Yes	Yes	Yes	No
**Secondary outcomes**						
	Psychological distress	K6^b^	Yes	Yes	Yes	No
	COVID-19 fear	Fear of COVID-19 Scale	Yes	Yes	Yes	No
	The total number of sickness absence days in the past 3 months	An original item	Yes	Yes	Yes	No
	Major depressive episodes^c^	MINI^d^ depression section: self-report	Yes	No	Yes	Yes

^a^BDI-II: Beck Depression Inventory II.

^b^K6: Kessler psychological distress scale.

^c^While major depressive episodes will be measured at 6- and 12-month follow-ups, the information will be combined to create a variable for major depressive episodes during the 12-month follow-up.

^d^MINI: Mini-International Neuropsychiatric Interview.

#### Primary Outcomes

The primary outcomes of the study are depression and psychological distress at 3- and 6-month follow-ups. Depression will be measured with the Japanese version of Beck Depression Inventory II (BDI-II) [46,47], a 21-item self-report inventory of depressive symptoms in the past 2 weeks, with each item to be scored from 0 to 3. The total scale score will be calculated and used as a measure of the severity of depressive symptoms.

#### Secondary Outcomes

The secondary outcomes include psychological distress, fear of COVID-19, sickness absence days at 3- and 6-month follow-ups, and MDEs at the 12-month follow-up. Psychological distress will be measured with the Japanese version of Kessler’s psychological distress scale (K6) [48,49], a six-item scale of psychological distress (depression and anxiety) in the past 30 days, with a response option range from 0 (none of the time) to 4 (all of the time). The total scale score will be used as an indicator of the degree of psychological distress. Fear of COVID-19 will be measured with the Japanese version of the Fear of COVID-19 Scale (FCV-19S) [50,51], which is a seven-item scale to measure fear reactions to the COVID-19 infection. Each item is rated on a 5-point scale (1-5), and the total score (7-35) will be used as the measure of the degree of fear of COVID-19. Sickness absence days in the past 3 months will be measured using the following single-item question [37]: “How many days did you miss an entire work day because of problems with your health during the past 3 months? (please include only days missed for issues with your own health, not someone else’s health.).” The presence of a MDE will be measured with a self-report scale developed based on the MINI [52] according to the Diagnostic and Statistical Manual of Mental Disorders-5 criteria [53]. The same set of nine questions from the MDE section of the MINI will be used to assess whether a participant has had a MDE in the past 12 months, followed by questions on the onset and the most recent time the participant has had symptoms of a MDE in the past 12 months. The sensitivity and specificity of this instrument for the clinical diagnosis of major depression were reported as 0.86 and 0.67, respectively, in a sample of psychiatric outpatients (n=31) in a pilot study. The self-report MINI for MDEs will be measured at 6- and 12-month follow-ups, as well as at baseline. Combining participants’ responses to these two follow-up surveys, any MDE reported after baseline and the timing of onset (number of months since baseline) will be used as the MDE outcome during the 12-month follow-up period.

### Process Evaluation

Information on the usage of the intervention program by participants in the intervention group will be collected from the records of the program. Questions will be asked in the baseline and follow-up questionnaires to gather information about respondents’ self-reported knowledge and self-efficacy regarding stress management in general, as well as the CBT components of the program (cognitive restructuring, behavioral activation, assertive communication, problem solving, and relaxation training) as follows: for knowledge, “how much knowledge do you have about…” and for self-efficacy, “how confident are you that you can do….,” with a 5-point scale ranging from 0 (none) to 4 (enough) [18].

### Implementation Outcomes

Implementation outcomes will be measured with the user version of the Implementation Outcome Scales for Digital Mental Health (iOSDMH) (unpublished study "Implementation Outcome Scales for Digital Mental Health (iOSDMH): a scale development and cross-sectional study" by Sasaki et al, 2021), a 19-item scale, with a 4-point Likert-type response option, as follows: three items for acceptability, four items for appropriateness, six items for feasibility, five items for harms, and one item for overall satisfaction. Participants in the intervention group will be asked to complete the questionnaire online at the 3-month follow-up. Participants in the intervention group will also be asked additional questions regarding the reasons for not participating in the program or discontinuing the program if they do so.

### Demographic and Other Characteristics

Demographic data, such as sex, age, education, marital status, occupation, type of employment contract, type of work shift, frequency of remote work (working from home), treatment of chronic physical conditions and mental disorders, and overtime hours during the past month, will be collected.

### Sample Size Calculation

The required sample size was calculated for one of the outcome variables, ie, depressive symptoms assessed by BDI-II. Previous meta-analyses of web-based universal prevention psychological interventions for improving depression and anxiety in the workplace yielded a summary effect size of 0.25 [8]; our previous study of a guided universal prevention iCBT program among workers reported a smaller effect size on depression (*d*=0.16) at a 6-month follow-up [18]. To detect a minimal effect size of 0.15 at an alpha of .05 and a power of 0.80, the estimated sample size is 699 participants in each group. The statistical power was calculated using the G*Power 3 program [54].

### Randomization

Participants who meet the inclusion criteria will be randomly allocated to the intervention or control group, as well as stratified into two groups based on BDI-II scores at baseline (≥14 or <14) [46]. An independent biostatistician will generate a stratified permuted block random table by using SAS (SAS Institute Inc). The stratified permuted-block random table will be password protected and blinded to the researchers, and sent to the CRC by an independent research assistant. The assignment will be conducted by the CRC.

### Statistical Methods

#### Effectiveness of the Intervention

For the primary and secondary outcomes, except for MDEs, a mixed model analysis for repeated measures will be used to test the intervention effect (group × time interactions) for 3- and 6-month follow-ups in the total sample (universal prevention), on an intention-to-treat basis. This model will handle and impute missing data with restricted maximum likelihood estimation assuming missing values at random. Effect sizes (Cohen *d*s) and 95% CIs at 3- and 6-month follow-ups will be calculated among those who complete the baseline and follow-up surveys. For MDEs at the 12-month follow-up, a Cox proportional hazard model will be used to estimate the preventive effect of the intervention program on MDEs. All statistical analyses will be conducted using SPSS Statistics v26.0 (IBM Corp).

#### Subgroup Analysis

The effectiveness of the program may be greater for participants with depression at baseline. We will conduct similar mixed model analyses for the effectiveness of the program among a subgroup of symptoms of depression at baseline (with a BDI-II score of ≥14; indicated prevention).

### Data Monitoring

A data and safety monitoring board will consist of a chair and two members, independent of the research team. The board will meet every 3 months after the first participants are randomized. The purpose of the meetings will be to review the report prepared by the CRC to monitor recruitment progress and data collection.

### Ethical Considerations

The researchers carefully developed the aims, design, and specific procedures of the study, and submitted a research application to the Research Ethics Review Board of the Graduate School of Medicine/Faculty of Medicine, The University of Tokyo. The board approved the application after a careful review and an interview with the researchers (number 3083-(6)). Informed consent will be obtained by the CRC from all participants included in this study after full disclosure and explanation of the purpose and procedures of the study. Candidate participants will be informed that their participation is totally voluntary, that even after voluntarily participating they can withdraw from the study at any time without stating the reason, and that neither participation nor withdrawal will cause any advantage or disadvantage to them. We expect no adverse health effects from this intervention, except possibly slight deterioration in depressive/anxiety symptoms [11]. We will provide an emergency phone number and email address at the research office. A research assistant will deal with the emergency calls or emails first and then consult with the clinical supervisor (NK) to provide appropriate care.

### Data Confidentiality

Participants will complete baseline and follow-up online questionnaires on a specially designed website. Researchers will not know any personally identifying information about them; only the CRC will know this information. Collected data from the questionnaires will be anonymized, linked, and stored in a password-locked file by the CRC and sent to the researchers at the University of Tokyo (NK, KW, KI, and NSasaki) for further analysis.

## Results

At the time this paper was submitted, the study was at the stage of recruitment of participants. The analysis of data will begin in January 2022 for the outcome variables, expect MDEs, and in July 2022 for MDEs. We expect to publish the results in 2022 or 2023.

## Discussion

### Strength of the Study

The strength of this study is its investigation of the effectiveness of a fully automated machine-guided iCBT for improving the subthreshold symptoms of depression among workers using an RCT design. The machine-guided iCBT is designed to incorporate several functions that were achieved by therapist support in previous guided iCBT programs [18], but are achieved by applying regression models, a scenario-based chatbot, and deep-learning technologies. The machine-guided supports would enhance the effects of an iCBT program on depression alleviation and other outcomes to a level equivalent to or greater than that for guided iCBT programs.

Exercises after lectures (or homework assignments) are an important part of CBT, and they help participants practice skill-based knowledge learned in the session [55]. The present machine-supported iCBT provides participants with opportunities to practice cognitive restructuring skills guided by the AI algorithm, instead of a therapist. More specifically, the algorithms will be used to train participants to correctly distinguish concepts of the five-part CBT model, such as events, thoughts, moods, physical symptoms, and behavioral reactions. Another important CBT component of the present iCBT program is behavior activation, where practicing skills in real life is important for improving negative mood [21,22]. In the exercise for Module 3, a step-by-step procedure guides participants to list candidate behaviors, reconsider avoidance behaviors, select behaviors for practice in real life, set up a behavioral activation plan, and review and revise the plan afterwards, just like a procedure taken by a therapist [43,56], where machine-guided functions help participants to complete each step. These functions would encourage participants to practice behavior activation skills more frequently and intensively compared to an unguided self-help iCBT program where participants only read instructions on how to make a behavior activation plan [57].

The other function included in the present iCBT program is self-assessment of work-related stressors and emotional states, which also gives participants future projections of work-related and personal life–related outcomes based on the results of the assessment (Module 1), using regression models developed based on a large database. This would not only help participants to understand the concepts of the components of a stress model, but also increase their awareness of their own stress levels and motivate them to engage in the program. This may enhance the effectiveness of the present iCBT program on depression alleviation by enhancing participants’ engagement in the program [58] or through improved self-monitoring for stress [36].

### Dissemination of the Findings

A fully automated machine-guided iCBT program requires less involvement of mental health specialists such as psychologists. It can be provided at a low cost in a low-resource setting in which trained practitioners are seldom available. Thus, the present fully automated machine-guided iCBT program has a lot of potential for dissemination as a practical tool for the prevention of depression in small- and middle-sized enterprises, and also in the workplace in low- and middle-income countries that do not have a well-organized training system for CBT counselors. Once we find the machine-guided iCBT sufficiently effective, it will contribute to the dissemination of a stress management program to a large number of workers to improve depression online at a low cost in the COVID-19 era where close social contact is limited.

The main findings of this study will be disseminated via publications in peer-reviewed international journals. Study findings will also be presented at scientific conferences. If the present program is found to be effective, a future plan to disseminate the program to a large number of workers in the workplace will be discussed with the government, nonprofit organizations, and corporations.

### Limitations

The major weaknesses of this study include that the study will not directly compare the effect of the machine-guided iCBT with that of a therapist-guided iCBT. While we can compare the effect size obtained from this study with past ones (Cohen *d*=0.16 to 0.25) [8,18], the comparison may be biased by differences between the studies in terms of the characteristics of participants, timing of the study, and other situational factors. The other weakness is that the machine-guided program developed in this study is not fully featured in terms of AI technologies. For instance, the program does not have a function to conduct natural conversations between a user and the system; additionally, the program is programmed based on machine learning of big data, but once fixed, the program will not learn any more to optimize the algorithm. The other problem related to the study design is that we will test the effectiveness of the program in healthy full-time workers. Thus, the study will not provide evidence for part-time workers. The study will not consider differences in work-related characteristics and major sources of stress among occupations. However, the study will provide a preliminary, but necessary, step toward establishing a machine-guided iCBT approach for preventing depression.

### Conclusion

This is the first study to investigate the effectiveness of a fully automated machine-guided iCBT program applying AI technologies for the improvement of the symptoms of depression among workers, using an RCT design. The study will explore the potential of the machine-guided stress management program that can be disseminated online to a large number of workers with minimal cost in the post–COVID-19 era.

## Appendix

Multimedia Appendix 1 Demonstration of the SMART-CBT program.

## Abbreviations

AI: artificial intelligence
BDI-II: Beck Depression Inventory II
CBT: cognitive behavioral therapy
CRC: clinical research coordinator
iCBT: internet-based cognitive behavioral therapy
LSTM: long short-term memory
MDE: major depressive episode
MINI: Mini-International Neuropsychiatric Interview
RCT: randomized controlled trial
TAU: treatment as usual
